# Multi-resolution visualization and analysis of biomolecular networks through hierarchical community detection and web-based graphical tools

**DOI:** 10.1371/journal.pone.0244241

**Published:** 2020-12-22

**Authors:** Paolo Perlasca, Marco Frasca, Cheick Tidiane Ba, Jessica Gliozzo, Marco Notaro, Mario Pennacchioni, Giorgio Valentini, Marco Mesiti

**Affiliations:** 1 AnacletoLab, Department of Computer Science, University of Milan, Milan, Italy; 2 Neuroradiology Unit, IRCCS San Raffaele Hospital, Milan, Italy; 3 CINI National Laboratory in Artificial Intelligence and Intelligent Systems—AIIS, Rome, Italy; Unviersity of Burgundy, FRANCE

## Abstract

The visual exploration and analysis of biomolecular networks is of paramount importance for identifying hidden and complex interaction patterns among proteins. Although many tools have been proposed for this task, they are mainly focused on the query and visualization of a single protein with its neighborhood. The global exploration of the entire network and the interpretation of its underlying structure still remains difficult, mainly due to the excessively large size of the biomolecular networks. In this paper we propose a novel multi-resolution representation and exploration approach that exploits hierarchical community detection algorithms for the identification of communities occurring in biomolecular networks. The proposed graphical rendering combines two types of nodes (protein and communities) and three types of edges (protein-protein, community-community, protein-community), and displays communities at different resolutions, allowing the user to interactively zoom in and out from different levels of the hierarchy. Links among communities are shown in terms of relationships and functional correlations among the biomolecules they contain. This form of navigation can be also combined by the user with a vertex centric visualization for identifying the communities holding a target biomolecule. Since communities gather limited-size groups of correlated proteins, the visualization and exploration of complex and large networks becomes feasible on off-the-shelf computer machines. The proposed graphical exploration strategies have been implemented and integrated in UNIPred-Web, a web application that we recently introduced for combining the UNIPred algorithm, able to address both integration and protein function prediction in an imbalance-aware fashion, with an easy to use vertex-centric exploration of the integrated network. The tool has been deeply amended from different standpoints, including the prediction core algorithm. Several tests on networks of different size and connectivity have been conducted to show off the vast potential of our methodology; moreover, enrichment analyses have been performed to assess the biological meaningfulness of detected communities. Finally, a CoV-human network has been embedded in the system, and a corresponding case study presented, including the visualization and the prediction of human host proteins that potentially interact with SARS-CoV2 proteins.

## Introduction

The analysis and interpretation of relationships between biological molecules and related concepts is becoming a major bottleneck in systems biology. Typically the pure amount of data and their heterogeneity and large size provide a challenge for their visualization. Biological entities, like proteins, are often represented through networks consisting of nodes, denoting the individual bio-entities, and edges, describing connections between nodes [1]. Interaction networks are one of the primary visual metaphors for communicating and understanding -omics data at a systems level. Several methods for the visualization of biomolecular networks have been recently proposed (see e.g. [2–4]). Nevertheless, as the number of entities and the interactions among nodes of different types (e.g. physical or genetic interactions) grows, the resulting networks are often complex and of too large size for a global visual representation. Moreover, facilities for the visualization and exploration of protein networks at multiple levels of resolution can be also required.

To contribute to fill this gap, this work presents a network visualization methodology to render and explore biological networks using different resolution levels by exploiting communities of highly correlated proteins. The approach relies on a hierarchical community detection algorithm that decomposes the network into non-overlapping communities: the initial view of the network plots communities as meta-nodes, and their total inter-connections as meta-edges, allowing to detect further topological hidden structures in the network. Communities of nodes highly connected and possibly sharing a common biological function (e.g. belonging to the same pathway or to connected pathways) can be thereby highlighted; furthermore, beside standard node-node connections, node-community connections are also displayed, for pointing out communities more related to a given target protein. By breaking down massive networks into smaller sub-networks, having a more clear topology, the visualization can guide the user towards unveiling the underlying biological mechanisms. Furthermore, a hierarchical decomposition is built, so that the navigation can explore individual meta-nodes, visualize their sub-communities, in a top-down fashion, till the most fine-grained one is reached, and meta-nodes are expanded to visualize the nodes they contain. Thus, node neighborhoods might be split by the recursive partition algorithm, so as to avoid the limitation of ‘classical’ *vertex-centric* visualizations. The user can also squeeze the expanded communities in a bottom-up manner, to make the exploration bi-directional. We named this novel visualization methodology *community-based navigation*, and embedded it in the UNIPred-Web service, recently introduced as one of the state-of-the-art tools for integrating biological networks in a user-customizable setting, and to predict the Gene Ontology protein functions [5] (https://unipred.di.unimi.it).

Protein function prediction is one of the central problems in Computational Biology, as witnessed by the CAFA international challenges [6–8]. Differently from other web-tools proposed for the same task, UNIPred-Web integrates protein networks and predicts protein functions by expressly considering the imbalance in protein labels (proteins annotated for specific functions are usually outnumbered by unannotated ones). Technical details of the integration [9] and inference algorithms [10, 11] have been published separately. The tool allows us to select the networks to be integrated from a repository of more than 1900 biomolecular networks of nine different organisms. In addition it also supports the inference of novel putative memberships to Gene Ontology (GO) terms [12] of genes and proteins belonging to the integrated network. Both integrated networks and predictions can be downloaded from the web-server, in different file formats. UNIPred-Web supported only vertex-centric exploration, i.e. centered on a given target protein, that, although being the most appropriate to analyze the properties of a specific gene/protein [13], in some cases might not supply a global view of the integrated network and, for large and complex networks with high average node degree, this visualization could produce a black cloud of points difficult to analyse. We thereby coupled the vertex-centric with the community-based navigation proposed here, where the user can study the systemic properties of sets of related genes/proteins starting the navigation from a target protein, and then benefits from having a general overview of the integrated network, with multiple views at different granularity levels. Moreover, a CoV-human protein interaction network, recently proposed by Gordon et al. [14] and included in the BioGRID database [15], has been embedded in the system. Finally, to improve the quality of predictions, a procedure for the automatic tuning of the hyper-parameters of the inference algorithm has been implemented and included in the amended version.

Table 1 reports the main tasks supported by this enhanced version of the UNIPred-Web application. The realization of these tasks required both: *i*) the development of graphical facilities for the rendering of the integrated networks; *ii*) indexing structures and algorithms for the preparation of the integrated networks and the communities hierarchy; and, *iii*) interaction facilities for supporting the user in the specification of the integration activities and for making him aware of the processes that are executed on the server. Summarizing, this paper introduces the following main contributions:

- By means of task **T3** and of the developed indexing structures, large PPI networks can be visualized at multiple levels of resolution;
- Through task **T2** a hierarchy of communities is detected and exploited for visualization by using the facilities developed for the tasks **T4** and **T5**;
- By means of task **T1**, the community-detection approach is integrated with semi-supervised protein function prediction and data integration algorithms to support explorative and predictive analysis of biomolecular networks. Since this is a time-consuming operation, an estimation of the time required for the integration is reported;
- The multi-level view made available through task **T3** has been combined with the vertex-centric visualization facilities (already available) in order to explore the community hierarchy from a target protein. Therefore, the new web tool can interactively navigate biomolecular networks using both a vertex-centric and a community-based exploration approach (task **T6**);
- Tasks **T7**, **T8**, and **T9** supply complementary facilities that support the user in the selection of the preferred protein ID mapping to export single communities of the integrated network, and monitor the status of the server depending on the assigned workloads;
- Case studies that show the effectiveness of the proposed methodology for the analysis of cancer and COVID-19 data.

**Table 1:** Main tasks supported by the proposed UNIPred-Web upgrade.

task id	task name	description
**T1**	**integration/prediction**	Specification of the experiment setting: networks to be integrated, proteins whose function should be inferred, type of visualization
**T2**	**hierarchical community detection**	Identification of the hierarchy of communities in the integrated network and construction of its visual representation
**T3**	**multi-level views**	Visualization of the integrated network at different resolution levels according to a community hierarchy
**T4**	**zoom in**	Starting from a meta-node in the current multi-resolution visualization, expands the visualization with its content
**T5**	**zoom out**	Starting from a node in current multi-resolution visualization, collapses the node in its most specific community along with all the other nodes belonging to the same community
**T6**	**combination of vertex and community-based exploration**	Integration and easy switching between protein-centric and community-based visualization and exploration
**T7**	**protein ID mapping**	Selection of the protein identifiers mapping
**T8**	**export**	Export a single community of proteins
**T9**	**server status**	Report the current workload level of the server and user active processes

### Related literature

#### Community-based network visualization and exploration tools

Many approaches have been proposed for the proper visualization and interactive analysis of complex graphs [16] and for the proper design of visualization and navigational tools [17, 18]. The key issue in the realization of these systems is the size of the network to be visualized. Indeed, when the graph size largely grows, the performances of the visualization facilities turn out to be unacceptable and a cloud of nodes is drawn making impossible to discern its content. Different clustering/community detection (CD) approaches have been proposed to reduce the number of visible elements and thus improving the clarity of the visualization and the performance of the visualization facilities [19]. These algorithms have been widely used to study the structure of complex networks and to unveil further levels of organisation at an intermediate scale. The task is to identify subset of nodes (communities or clusters or groups or modules) more densely interconnected with one another than with the remainder of the network. Although no formal definition of community is universally accepted [20], a largely adopted measure to quantify the quality of communities is the minimization of *modularity function* [21], allowing partitioning nodes into communities such that nodes within a community are more likely to connect to one another than expected in a random network null model [22]. Globally optimizing the modularity is known to be a NP-hard problem [23], therefore usually some local heuristics are adopted [24–30], mostly based on greedy criteria.

When the clustering is hierarchical, the graph can be visualized according to the structure imposed by the hierarchy and navigation operations can be implemented for grouping and ungrouping communities [16]. In this way, a general overview of the graph is provided by means of the higher levels of the hierarchy, and further details of the graph can be obtained by descending the hierarchy [31]. Both agglomerative [24, 32] and divisive algorithms have been provided for this purpose [26], while more sophisticated techniques are based on the construction of a multi-layer network, where each layer has a dedicated scale parameter [33]. Divisive methods often poorly scales on large-sized networks, due to the computation of heavy measures to detect “hot” edges like the edge betweenness, whereas the approach [33] has too many (hyper)parameters to be tuned, making it impracticable for our purpose, having our web-interface the need of providing fast responses to user requests. Approaches and systems for the visual exploration of the hierarchical communities have been proposed [34, 35] as well as multi-resolution visualizations of cellular network processes [36] and biological pathways [37]. However, their focus is mainly centered on the rendering of the communities on the canvas, whereas our focus is on the data and indexing structures adopted for easily retrieving and preparing big graphs to be rendered.

#### Visualization and prediction web-tools for protein networks

In the last few years, a bunch of tools for predicting protein functions and exploring protein networks have been designed, often supporting also their integration. Table 2 reports the most representative web-based approaches that offer an interactive exploration of the protein networks (many others can be found in [1]). For each tool, we report: its website; the adopted visualization library; the presence of a database of imported biological networks from experimentally derived protein–protein interactions available on the Web (db), or if it can work only with external data (ext) that is uploaded on-demand or both; the possibility to integrate networks in a custom way (on demand) or in a fixed setting (pre-def); the availability of tools for protein function prediction; the identification of communities/clusters of similar biomolecules; and the kind of visual exploration and navigation of the network shown on the screen: a vertex-centric approach (vc) in which the target protein is shown with its neighbour; the possibility to expand the visualization either based on a maximum number of nodes to display (exp) or starting from any node of the network (exp^⋆^); and a navigation that exploits the identified communities/cluster of biomolecules (com).

**Table 2:** A comparison of visual tools for the exploration of biomolecular networks.

Tool	website	Vis. Lib.	Data	Integration	GO term Prediction	Clustering	Navigation
Genemania [2]	link	cytoscape	both	pre-def	no	yes	vc,exp
PINV [39]	link	BioJS	ext.	no	no	under dev.	vc,exp
ZoomOut [40]	link	sigma.js	ext.	no	no	yes	vc
UniHI [41]	link	cytoscape	db	pre-def	no	no	vc
Mentha [42]	link	SPV	db	pre-def	no	no	vc,exp^⋆^
STRING [3]	link	cytoscape	db	pre-def	no	yes	vc,exp,com
IMP 2.0 [43]	link	not spec.	db	pre-def	yes	no	vc,exp
UNIPred-Web [5]	link	cytoscape	both	on-demand	yes	yes	vc,exp^⋆^,com

The issue of exploiting a hierarchical community-based exploration of protein networks at the visual level is currently addressed only by UNIPred-Web 2020 (that builds on the ideas presented in [38]) and STRING. Nevertheless, UNIPred-Web 2020 shows communities of nodes at different levels of detail and can expand or collapse community on-demand. On the contrary STRING can just cluster nodes by coloring them, but without giving the user the possibility to expand or collapse the displayed communities. Moreover, the combination of the vertex-centric and community-based exploration facilities are a key characteristics of UNIPred-Web 2020 that is not available in other systems. ZoomOut can apply clustering methods using a set of computed descriptors for each network and all networks can be visualized as single nodes of a super-network, were interconnections among networks are based on the calculated clustering distances. However, this tool does not identify communities/clusters inside each network but only clusters of networks, thus defining an alternative clustering concept with respect to the one adopted in UNIPred-Web.

## Results

### Network integration, community-based detection and GO prediction

**Fig 1:**
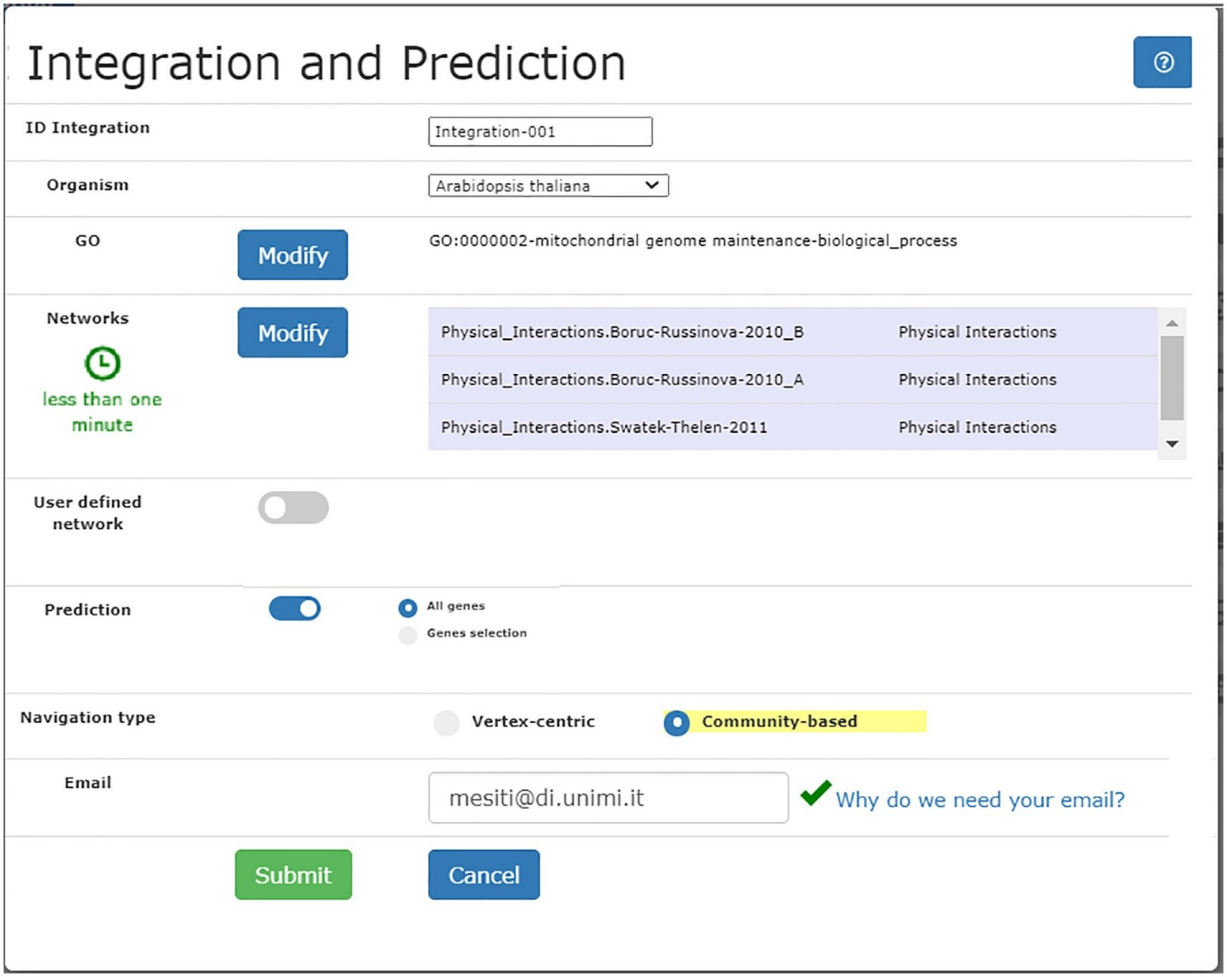
Specification of the networks integration/prediction and kind of navigation.

Integration and GO prediction represent one of the main tasks (task **T1**, Table 1) supported by our tool: Fig 1 shows the interface by which the user can specify the networks to be integrated, the GO term to be predicted, the use of external networks (optional), and the preferred kind of navigation. When the Community-based option is checked, after integrating networks through the UNIPred algorithm, a hierarchy of protein communities from the integrated network is created as shown in Section *Methods and Models*.

The network integration, the subsequent functional prediction, and the construction of the hierarchy of communities are realized at the server-side; although all the steps are realized through scalable procedures, the whole process can be time-consuming when integrating big networks. For this reason, an estimation of the time required for the integration is reported within the form. Moreover, a scheduler has been realized in the back-end for the management of the queue of experiment jobs. The scheduler considers the size of the networks to be integrated, the estimation for the time required for their computation, and the number of requests to be handled, so as to ensure that all user requests are satisfied.

Other facilities have been realized for supporting the users in the preparation of their experiments (Task **T9**). First, a semaphore reports the workload status of the server that takes into account the CPU and main memory occupation, the number of integration jobs that need to be scheduled, and the estimation of their execution times. In this way, users can take the decision to visualize other experiments or to wait on-line the end of the process. In any case, when the process is concluded, an email will be delivered to the user, containing the instructions for loading the integrated network and thus starting the navigation. Moreover, a log button is reported in the top right corner of the main interface by means of which they can see the integration jobs that: *i*) are currently processed by the server; *ii*) have been completed and the navigation to the associated networks can be started; and *iii*) have been removed from the server. In this way, the user is made aware of the activities that the server is doing (or has done) for him. All these ancillary functionalities associated with Task **9** support the user in the integration/prediction task.

### Community-based visualization and navigation

**Fig 2:**
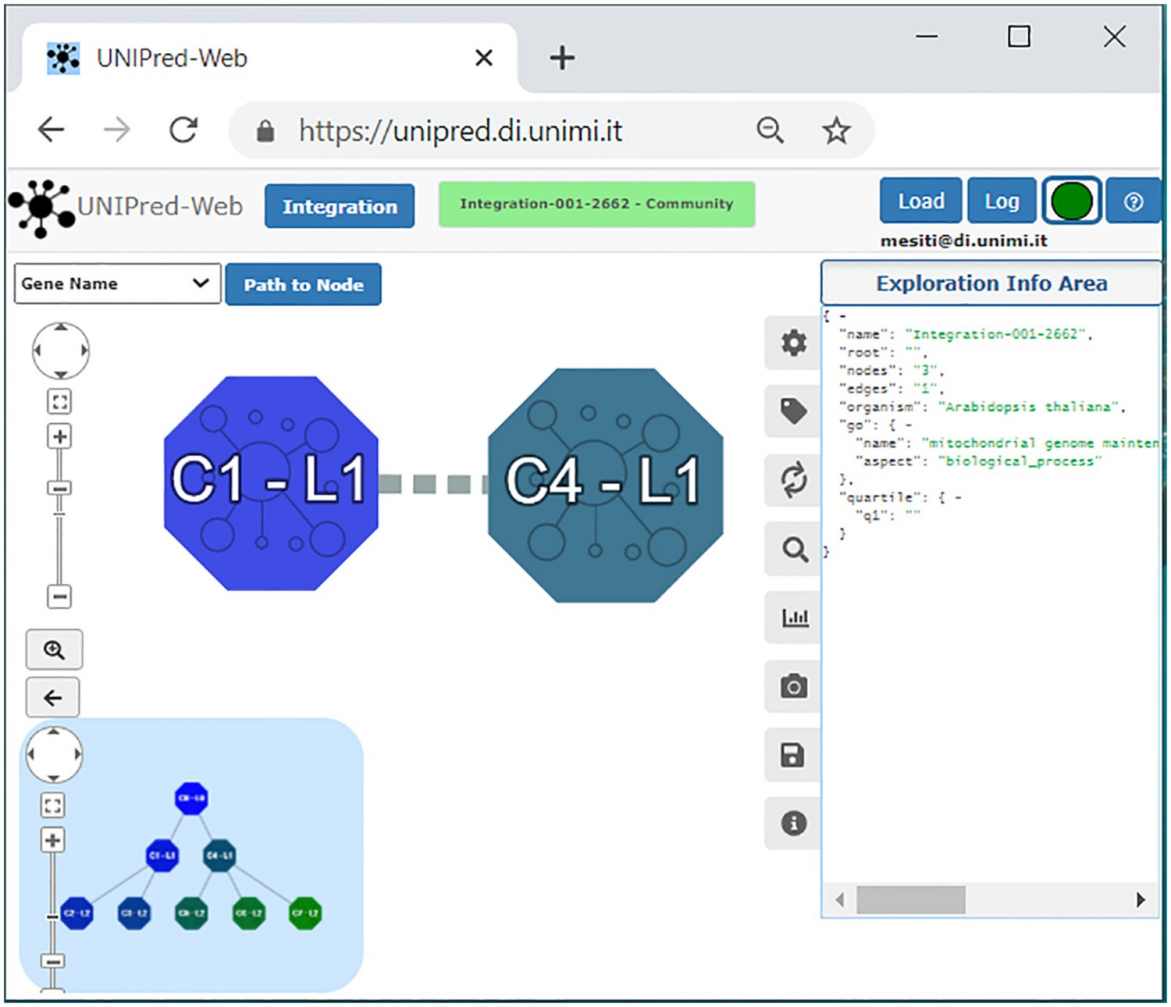
Starting point of the community-based navigation.

When the integration is completed, the user can start the community-based visualization and navigation of the integrated network (task **T3**). As shown in Fig 2, the communities at the first level (the one after the root) of the hierarchy are shown in the canvas. In this case, two communities are identified and labeled with C1-L1 and C4-L1 (L1 corresponds to the first layer in the community hierarchy). Each community is drawn with a different color and the size of the graphical object reflects the number of biomolecules that they contain (e.g. C4 contains more biomolecules than C1). Dashed edges can connect pairs of communities and they represent the existence of relationships between their biomolecules. The thickness of the dashed lines denotes the number of identified relationships. Popup panels associated with meta-nodes show information about the number of biomolecules and the kind of evidence from which they have been annotated; on the other side, by means of popup panels associated with the edge between the two communities, the user can read the number of relationships existing among biomolecules of the two communities along with the maximal, minimal and average weight associated with the relationships. Moreover, in the left bottom corner, a novel feature is also added for supporting task **T2**. A little canvas shows the *maptree*, that is a visual representation of the detected overall community hierarchy. The area where the maptree is located can be collapsed (for reducing space), or enlarged and interactively modified for making clearer the organization of the hierarchy. This novel feature provides a high-level representation of the hierarchical structure of the communities.

Another important novel feature in the network visualization is the possibility for the user to choose among a large set of protein/gene identifiers (task **T7**). Seven identification mapping are considered (see menu on the top left corner of Fig 2): gene name (official symbol), Ensembl gene ID, Ensembl protein ID, Entrez gene ID, Refseq protein ID, Refseq mRNA ID, Uniprot ID, and in addition existing protein aliases (synonyms). This information is shown even when inspecting the properties of a biomolecule through the associated popup and can be exploited also when downloading the integrated network, thus the user can receive the network in the identification scheme desired. It is worth pointing out that a correspondence among the Ensembl identification scheme and other schemes is not always available. In this case, the Ensembl identifier is used.

### Exploring the communities at multiple levels of resolution

**Fig 3:**
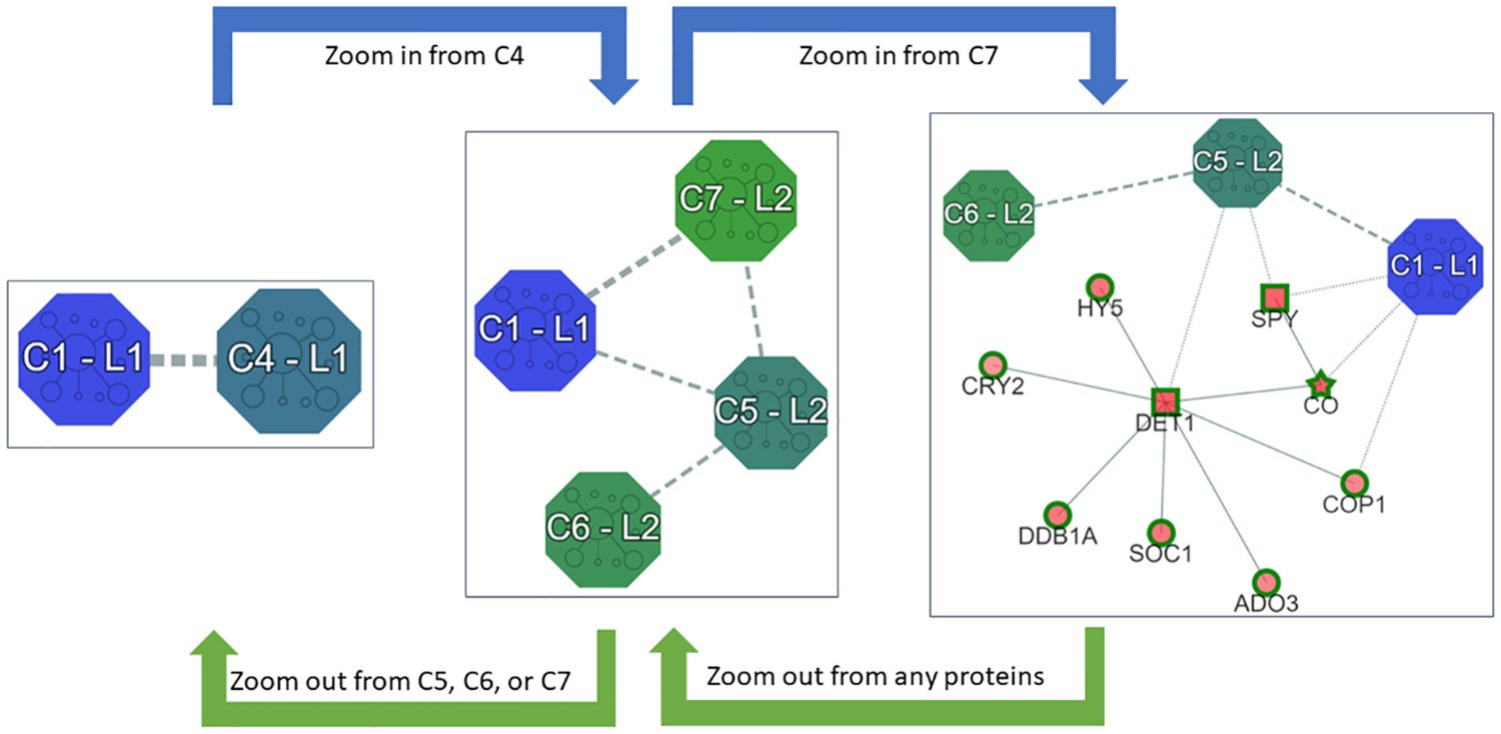
Zoom in/out of the multi-resolution representation of the integrated network.

By applying zoom-in and zoom-out operations, we can can explore the communities at multiple levels of resolution. For instance the user can choose the meta-node to be inspected and expand its content. The view obtained introduces the child communities of the selected meta-node in the visualization along with their relationships with the other meta-nodes and (eventually) the proteins currently present in the canvas. Moreover, the size of the communities is re-arranged to provide a comparison with respect to the size of the communities just introduced. For example, consider the multi-resolution representation of the biomolecular network reported on the left part of Fig 3, in which the communities C1 and C4 of the first layer are displayed, and suppose the user is interested in expanding the community C4. By selecting the *zoom in* option (task **T4**) among the operations that can be invoked on the meta-node, the community C4 is substituted by the communities C5, C6, and C7 (see the representation in the center of Fig 3). Furthermore, the relationships that exist among them and the community C1 are shown. The user can easily note that only the communities C5 and C7 present relationships with C1 and that C6 has relationships only with C5.

The community C7 is a leaf in the hierarchy (see the maptree in the left corner of Fig 2), and hence includes only proteins and no other communities. In this way, the canvas contains two kinds of nodes: those representing communities and those representing biomolecules (Fig 3). As a consequence, three kinds of edges can be identified: *solid lines* represent relationships among biomolecules, *dashed lines* relationships with communities, and *dotted lines* between communities and biomolecules. Moreover, the border of the biomolecules has the same color of the community C7, to highlight their membership to this community. We remark that, as reported in [5], when biomolecules are drawn as white circles, no prediction has been required for the integrated network. By contrast, when the prediction option is activated, nodes are internally colored with different gradation of red, reflecting the prediction score assigned to the biomolecule (the higher the score, the more intense the color provided). Moreover, their shape is: a) a *square*, when the biomolecules are annotated with the selected GO term; b) a *star*, when they are predicted to be annotated with it; c) a *circle*, otherwise.

As opposite, the zoom out operation (task **T5**) can be invoked on a single biomolecule or a meta-node to substitute it with its community (the same operation is simultaneously applied to the other nodes belonging to the same community). For example, by zooming out on the protein SOC1 in the right part of Fig 3, the visualization of the central part of Fig 3 is proposed. By further zooming out on one of the community C5, C6, or C7, we can move back to the initial visualization. The zoom in and zoom out are thus operations that allow us to easily navigate up and down in the community hierarchy, as depicted in Fig 3.

### Combining vertex-centric and community-based exploration

**Fig 4:**
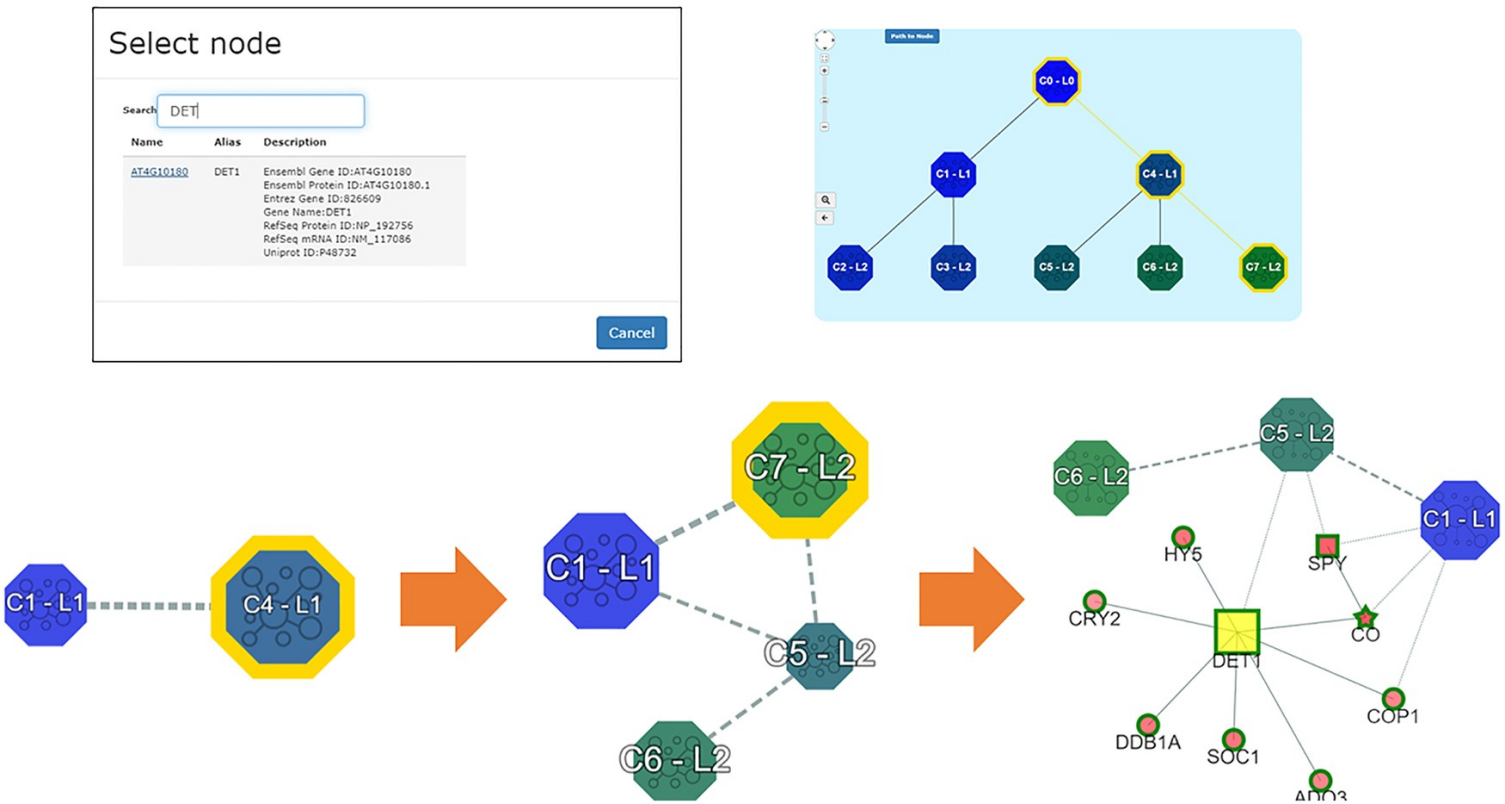
Combination of the vertex-based and community-based navigation.

The vertex-based exploration of the network, already available in the previous version of the system, can be now combined with the community-based exploration (task **T6**). The user can select at any point of the navigation a specific biomolecule to investigate and at the same time continue the exploration of the communities towards the leaf community containing it. The “path to node” button (left upper corner of the main interface) allows to select this modality. Once selected the biomolecule to be searched, the system highlights in yellow the border of the community that contains it. By zooming in, the user can expand the view till the lowest resolution is reached. Moreover, in the maptree, the corresponding path from the root to the leaf community is highlighted in yellow. This navigation option offers the possibility to identify relationships among the search node and the communities. For example, in Fig 4 the protein DET1 is searched, and the community C4 is highlighted. Then, zooming in C4, the community C7 is highlighted. Finally, by zooming in C7, the protein DET1 is marked.

### Exporting subnetworks of communities

**Fig 5:**
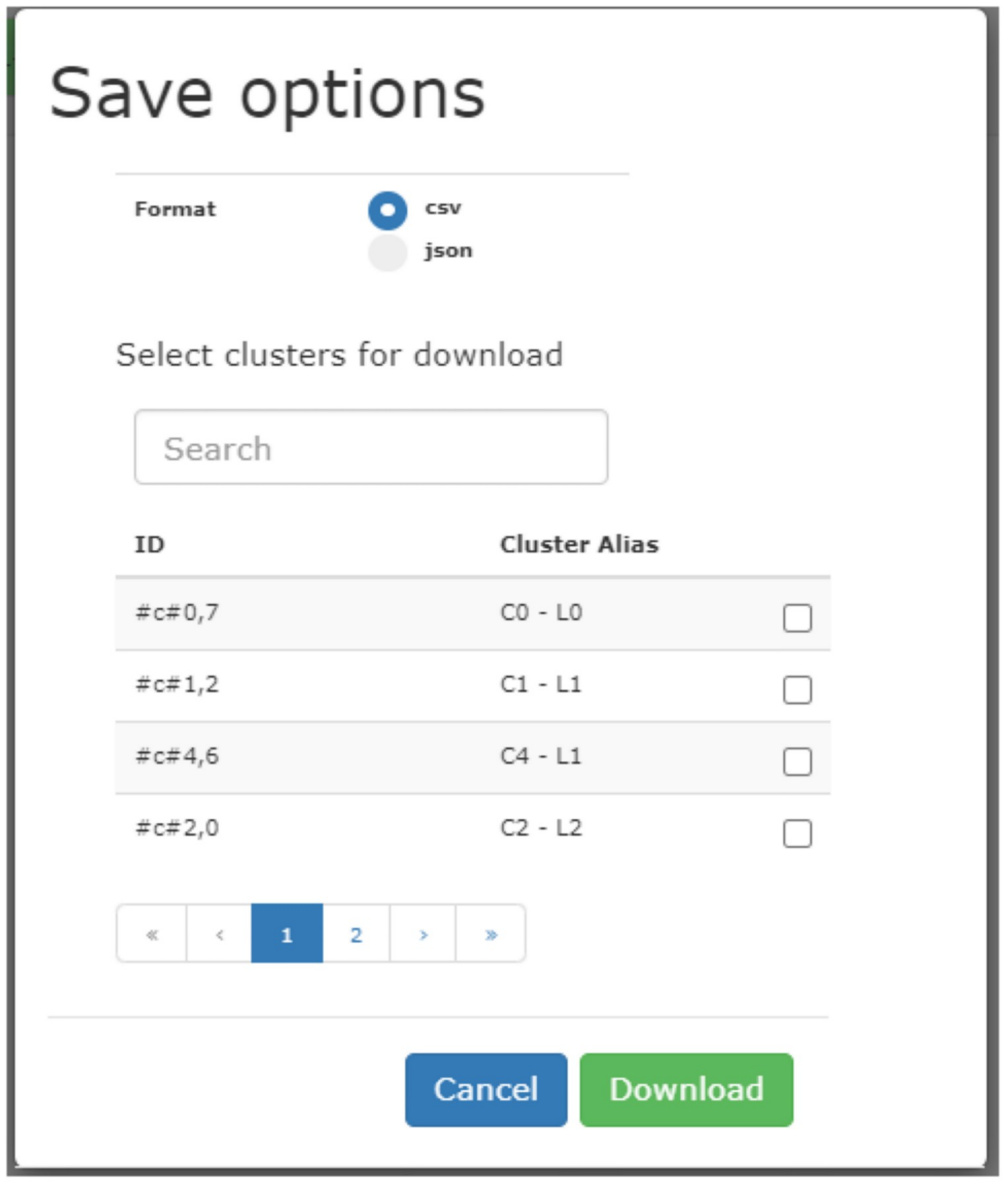
The option for exporting sub-networks according to the detected communities.

Task **T8** provides the possibility of exporting the sub-networks included in one or more communities. In this way the user can extract portions of the integrated network that can be processed by other analytical tools. The “save data” button on the right panel displays the interface in Fig 5, to select and export communities/sub-networks in tsv or json format, along with prediction scores associated with its vertices. Hence the user can further analyze the exported data with other available tools, as shown in the following case studies.

### Case study 1: Cancer pathway networks

To assess the potentialities, the reliability, and the robustness of the novel hierarchical community detection introduced, two human pathway networks already provided in UNIPred-Web have been selected for a case study:

- Pathway.NCI_NATURE (|*V*|: 2126, |*E*|: 10122) [44]
- Pathway.CELL_MAP (|*V*|: 408, |*E*|: 598) [45]

The Cancer Cell Map (CCM) [45] contains cancer-related signalling pathways while the National Cancer Institute/Nature Pathway Interaction Database (PID) [44] is a curated and peer-reviewed collection of human molecular signaling and regulatory events and key cellular processes. We integrated these two networks with the goal of supplementing the general purpose PID database with additional signalling pathways from the CCM database, thus obtaining a final network suitable to study cancer-related pathways. We selected the biological process term GO:0038066 (p38MAPK cascade) for our analysis. Indeed, the p38 MAP kinase signalling pathway is known to be deregulated in different tumors [46, 47]. The achieved integrated network consists of 2255 nodes and 10673 edges.

The hierarchical algorithm returned a three layers hierarchy with 52 total communities and 42 of them were leaves. All the leaf communities have a number of proteins between 14 and 156 (mean 52.5), which is a fair community size to conduct further functional analysis. We sorted in a decreasing order the communities on the basis of the number of nodes, and we performed an over-representation analysis for KEGG pathways using the R package RDAVIDWebService [48] on the first 10. Moreover, we considered as enriched only the pathways with a Bonferroni corrected *p*-value <0.05. Table 3 reports for each community the top 3-enriched pathways with the corresponding literature evidence.

**Table 3:** Top 3-enriched pathways with literature evidence.

Community	Proteins in community	Enriched pathway	Bonferroni	Involved Genes / Total Gene	References
C30	156	TNF signaling pathway	3.23^−37^	24.16%	[49] [50]
		NF-kappa B signaling pathway	6.07^−34^	21.48%	
		Apoptosis	4.13^−32^	18.79%	
C31	148	hECM-receptor interaction	5.38^−57^	30.82%	[51]
		Focal adhesion	6.80^−48^	35.62%	
		PI3K-Akt signaling pathway	9.17^−36^	35.62%	
C19	103	Cell cycle	7.21^−03^	8.25%	[52]
		HTLV-I infection	2.20^−02^	10.31%	
C28	84	Axon guidance	1.18^−17^	23.46%	[53] [54]
		Sphingolipid signaling pathway	1.19^−05^	12.35%	
		Regulation of actin cytoskeleton	1.55^−04^	13.58%	
C7	80	Cell cycle	6.61^−08^	13.92%	[55]
		Progesterone-mediated oocyte maturation	1.28^−06^	11.39%	
		Ubiquitin mediated proteolysis	4.44^−05^	11.39%	
C11	80	TGF-beta signaling pathway	3.30^−35^	35.53%	[56]
		Hippo signaling pathway	1.13^−07^	17.11%	
		Signaling pathways regulating pluripotency of stem cells	1.02^−03^	11.84%	
C13	76	Wnt signaling pathway	1.19^−18^	26.67%	[57] [58]
		Basal cell carcinoma	7.78^−06^	10.67%	
		Melanogenesis	3.86^−05^	12.00%	
C20	69	Adherens junction	7.26^−06^	13.43%	[59]
		Cell adhesion molecules (CAMs)	1.38^−05^	16.42%	
		Regulation of actin cytoskeleton	3.71^−03^	14.93%	
C25	68	TNF signaling pathway	1.81^−05^	13.64%	[60]
		Osteoclast differentiation	1.21^−02^	10.61%	
C26	66	Cell cycle	9.21^−15^	27.42%	[61]
		Small cell lung cancer	8.15^−06^	14.52%	
		Hepatitis B	4.93^−04^	14.52%	

List of top-3 enriched pathways for each considered community with Bonferroni corrected *p*-*value* < 0.05. Literature references connecting the pathways found in the community are provided. For community C19 and C25 we show only the first top-2 enriched pathways since the third one was not statistically significant with a Bonferroni corrected *p*-*value* > 0.05.

The idea of applying enrichment methods to identify relevant subnetworks from a biological standpoint is a well-known approach performed in literature using: a) ontology-based enrichment analysis [62], b) *de novo* enrichment analysis [63], c) community-based algorithms followed by semantic rule induction [64] to link biological explanations to each discovered subgroups. Similarly, in our work we used community detection followed by enrichment analysis to dissect our biomolecular network in subgroups described by enriched pathways coherent and correlated inside each community. For instance let us consider the community C30, containing the KEGG terms TNF signaling pathway, NF-kappa B signaling pathway and Apoptosis. Literature evidence (reported in the last column of Table 3), confirmed the strong correlation among terms associated with the same community. Indeed, TNF signaling pathway mediates its pro-inflammatory response by activating NFK-kappa B, while by activating a caspase, induces apoptosis instead. Furthermore, sustained activation of NFKB inhibits apoptosis.

Thus the detected communities analysed resulted coherently enriched in the pathway enrichment analysis, suggesting a correlation/structure among nodes identified and emphasized by the CD algorithm and by the corresponding graphical visualization.

### Case study 2: CoV-human network

With the goal to help the scientific community in addressing the ongoing global health crisis related to the rapid spreading of the SARS-CoV2 infection [65], UNIPred-Web 2020 includes in the large set of already available networks a novel Human-virus protein interactions network, named *CoV-human*, recently proposed in [14] and retrieved from the BioGRID database [15] (3.5.185 release). *CoV-human* includes only physical (i.e. Affinity Capture-MS, Affinity Capture-Western, Biochemical Activity, Co-crystal Structure, Co-localization, PCA, Reconstituted Complex, Two-hybrid) interactions between human (taxonID: 9606) and three different viral strains, SARS-CoV (taxonID: 694009), SARS-CoV2 (taxonID: 2697049) and MERS-CoV (taxonID: 1335626). The *CoV-human* network includes 418 nodes and 412 edges, of which 123 nodes and 108 edges are found in *SARS-CoV-human* sub-network, 320 nodes and 298 edges in the *SARS-CoV2-human* and 9 nodes and 6 edges are found in the *MERS-CoV-human*. Since all interactions are of high quality (i.e. predicted interactions are not present), we considered all of them as equally informative unitary edges in the network tuple format. From the Gene Ontology Annotation (GOA) database [66] we downloaded the protein-GO term associations (May 2020 release) for the proteins present in the *CoV-human* network. For the three virus strains we considered all the annotation types provided by GOA (i.e. IDA, IEA, IEP, IMP, IPI). Instead, for the human organism we extracted just the experimentally supported annotations i.e. the annotations with the following experimental evidence codes: EXP, IDA, IPI, IMP, IGI, IEP, HDA, HEP, HMP. The full description of these experimental evidence codes can be found at http://geneontology.org/docs/guide-go-evidence-codes/. Since the GOA database provides annotations according to the UniprotAC identification scheme (the same that we used in our “CoV-human” network), we did not lose any annotated protein. Finally, we propagated the annotations by transitive closure obtaining an annotation matrix with 418 proteins and 4788 functional terms, by gathering all the three GO sub-ontologies: biological process (BP), molecular function (MF) and cellular component (CC).

**Fig 6:**
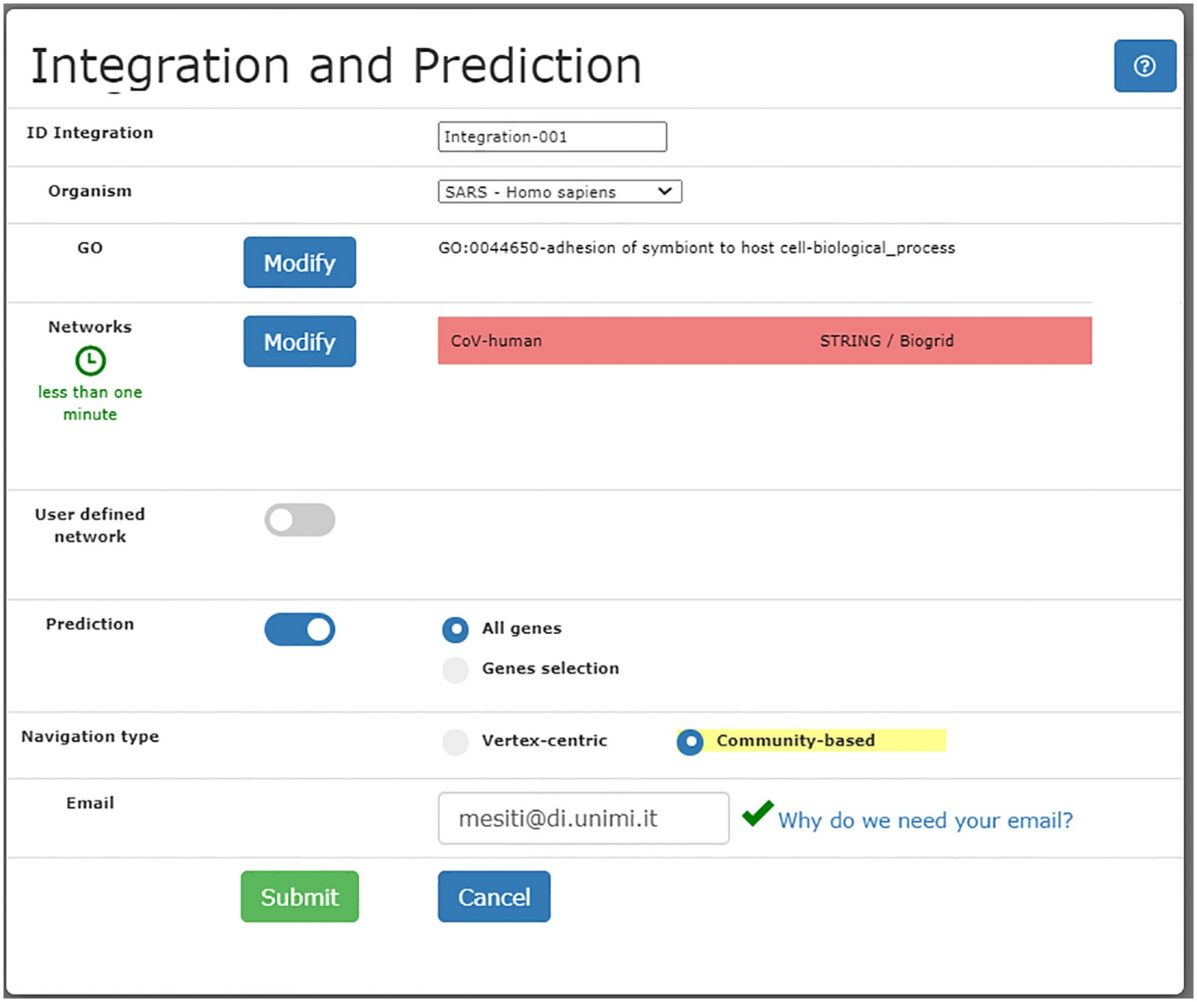
“Integration and prediction” panel for CoV-human protein-protein interaction network.

UNIPred-Web predicts a specific GO term for each protein in the network, aiding the process of finding new candidate targets for drug repositioning or novel insights about unknown disease mechanisms. For instance, suppose a researcher is interested in finding new proteins involved in the adhesion of the virus to the host cell surface, which could be candidate targets for drug repurposing to prevent cell infection. In UNIPred-Web the investigator can visualize the network and predict protein annotations for the GO BP term “adhesion of symbiont to host cell” (GO:0044650), whose description is “*The attachment of a symbiont to a host cell via adhesion molecules, general stickiness etc., either directly or indirectly*”. It is worth noting that this functional term was initially annotated only with three proteins. This intrinsic lack of information supports the application of bioinformatic tools (as UNIPred-Web) to predict potential protein-GO term associations. By means of the “Integration and Prediction” panel in Fig 6, the user can select the SARS-Homo sapiens as organism and the CoV-human as network and require the prediction of all proteins with respect to the term GO:0044650.

**Fig 7:**
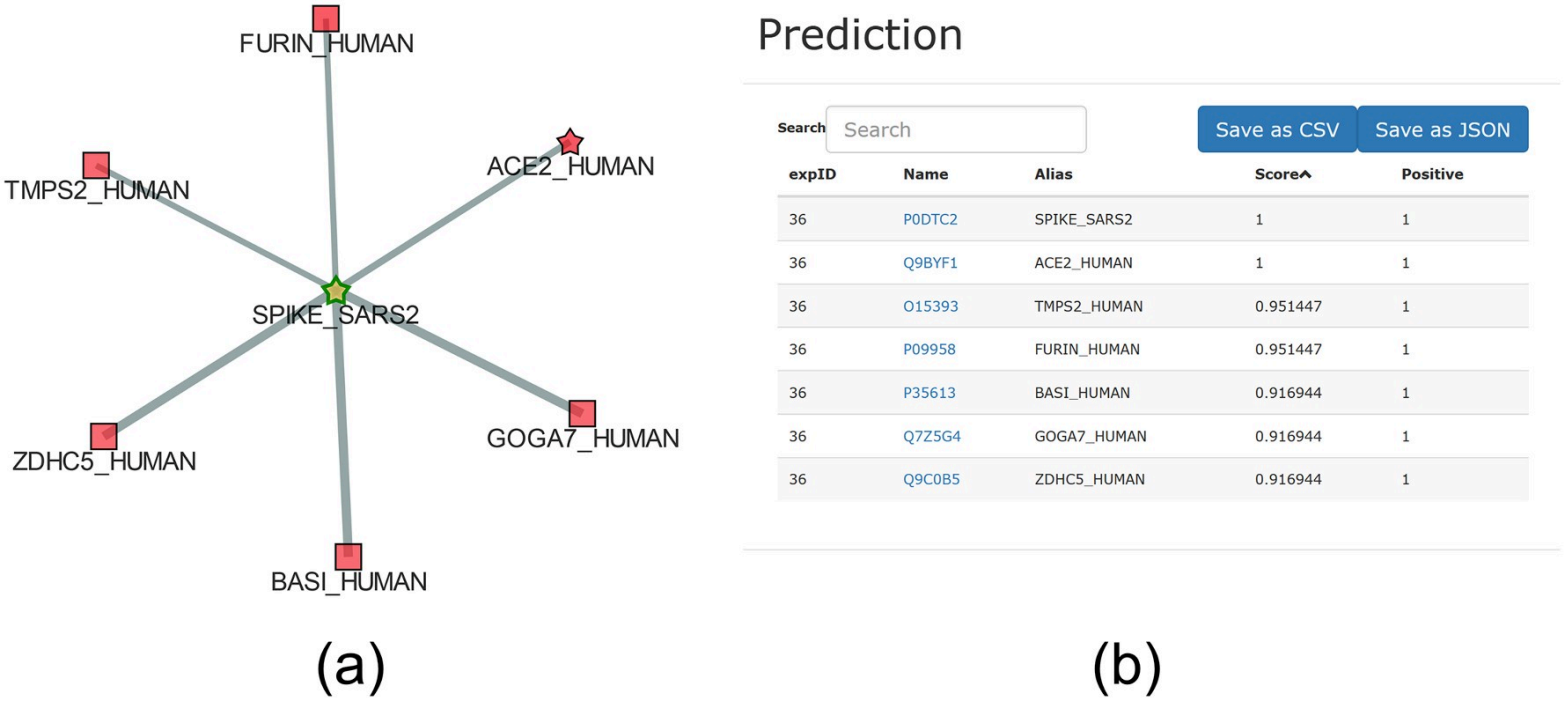
(a) CoV-human subnetwork centered on Spike glycoprotein (highlighted using “color shape settings” to obtain a better visualization). Stars represent proteins already known to be annotated for the GO term considered in our study (i.e. GO:0044650); squares represent proteins predicted to be annotated with a high score. The thickness of the links between proteins is proportional to the edge’s weight. (b) Prediction panel with scores sorted in a decreasing order for the subgraph shown in panel (a).

The visualization has been centered on the viral Spike glycoprotein (Fig 7), since it is known to promote the entry of virions in the host cells through the binding with the human receptor ACE2 [67].

**Fig 8:**
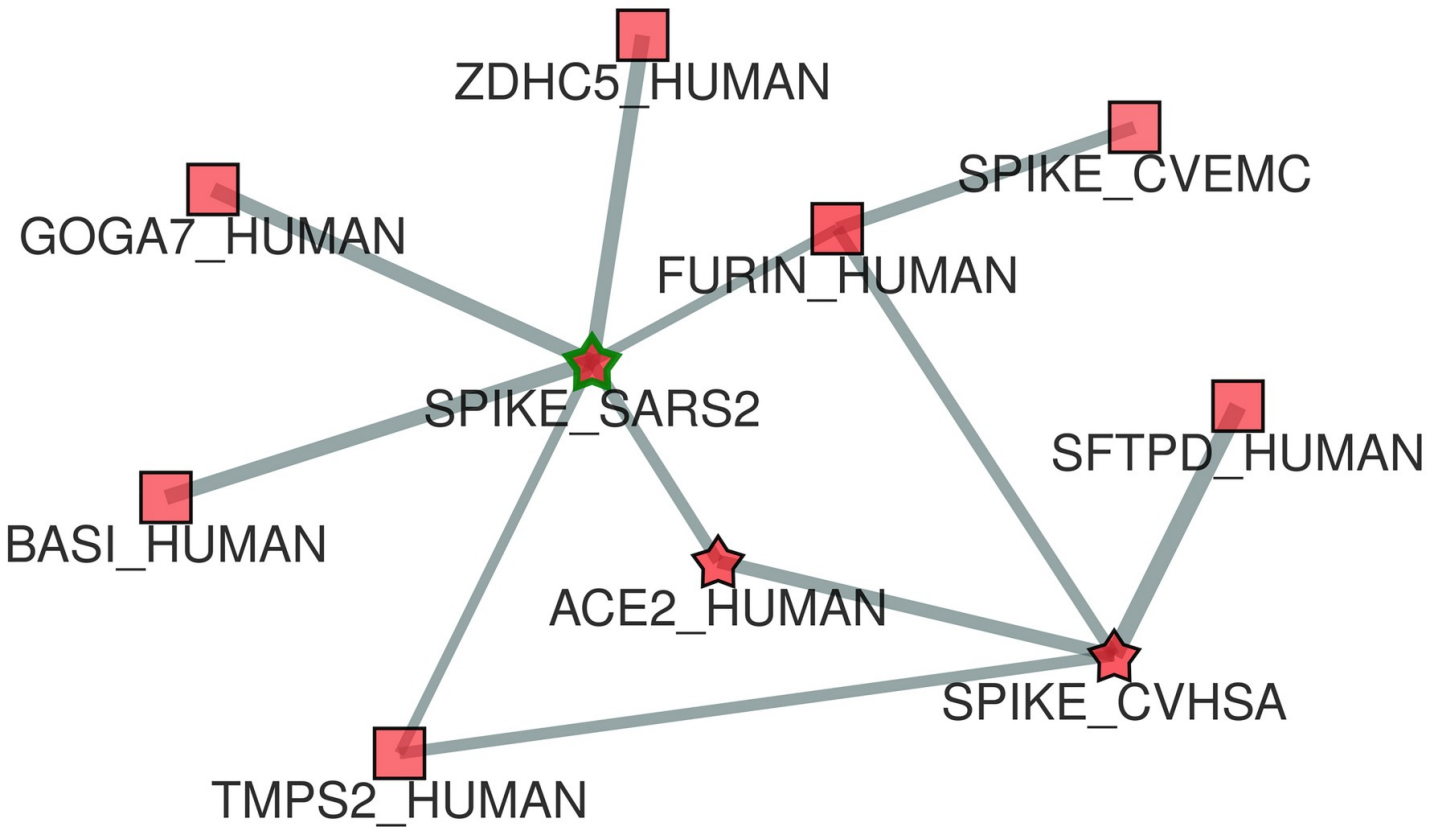
CoV-human subnetwork including all the 10 “positive” annotated or predicted proteins for the GO term considered in our study (GO:0044650). Stars refer to proteins already annotated and squares refer to proteins predicted and annotated with a high score. Thickness of the links between proteins is proportional to the edge’s weight.

The prediction scores of the network shown in Fig 7(a) can be visualized by opening the “Prediction panel” (Fig 7(b)). It is worth noting that the two viral proteins (SPIKE_SARS2 and ACE2_HUMAN) were already annotated with the considered functional term (GO:0044650) and are labelled with a star in the graph (Fig 7(a)). Instead, the other human proteins (TMPS2_HUMAN, FURIN_HUMAN, BASI_HUMAN, GOGA7_HUMAN, ZDHC5_HUMAN) were predicted to be annotated with the functional term GO:0044650 by UNIPred-Web (prediction score ≃1, the maximum, and labelled with a square in Fig 7(a)), which means they are strong putative candidate proteins for this GO term. Furthermore, always from the “Prediction Panel”, we can visualize the predictions for the whole network, which shows another predicted human protein SFTPD_HUMAN (score 0.97). By opening the popup panel associated with the node FURIN_HUMAN and exploiting the option ‘one step from here’, we can observe that this protein interacts with the viral protein SPIKE_CVHSA, which in turn interacts with the human protein SFTPD_HUMAN (Fig 8). Interestingly, the human surfactant protein D interacts with the Spike glycoprotein (S) of the viral strain SARS-CoV (taxonID: 694009), which in turn interacts with three different human proteins (TMPS2_H,UMAN, ACE2_HUMAN and FURIN_HUMAN), and these last ones interact with the Spike glycoprotein of the viral strain SARS-CoV2 (taxonID: 2697049). These ‘two-steps’ networks of interactions suggest that the Spike glycoproteins of the two SARS strains are closely related, as confirmed in [68].

**Fig 9:**
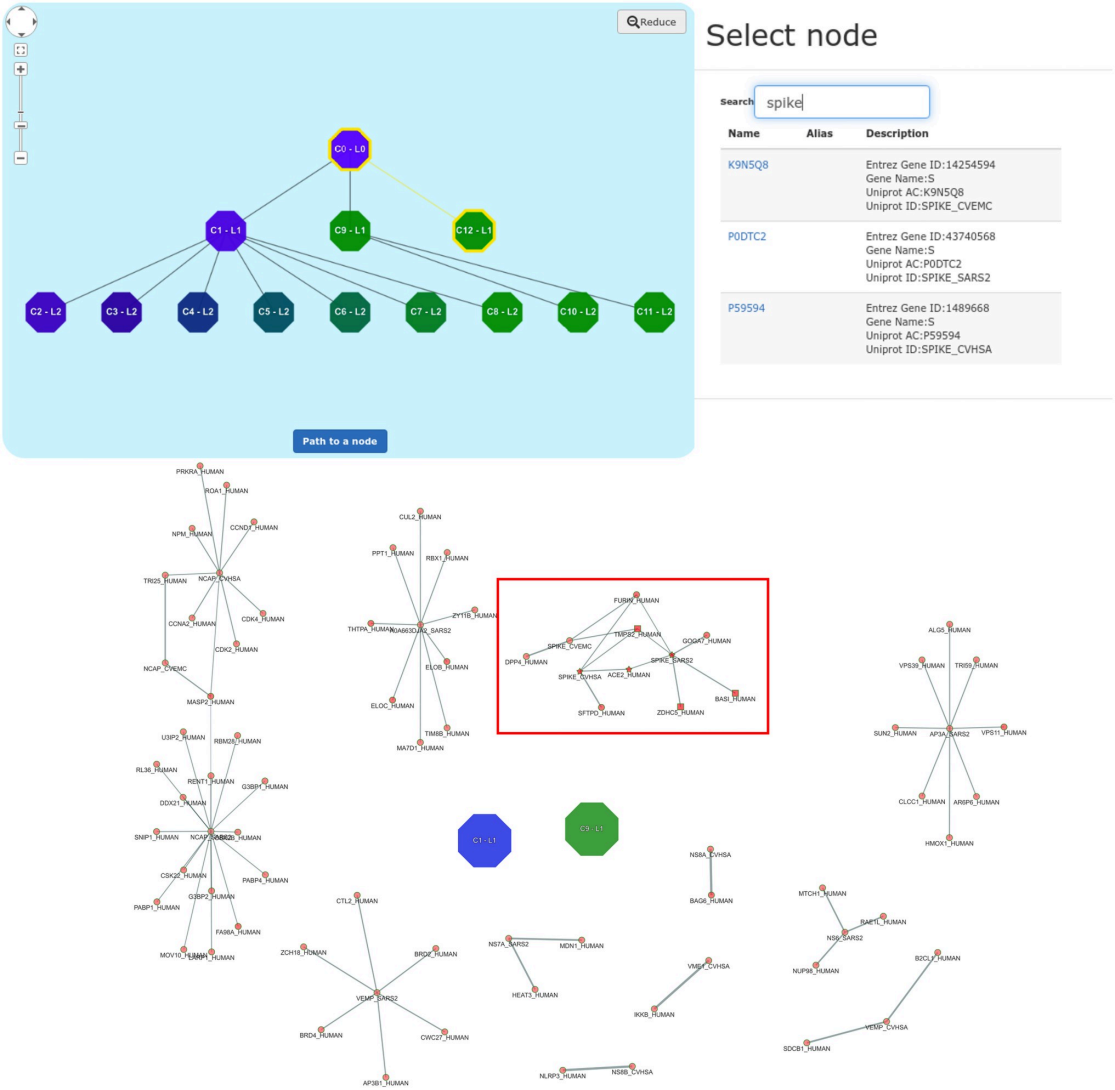
Hierarchical communities obtained from CoV-human network using the “community-based” visualization option provided by UNIPred-Web. The user can press the button “path to a node” and search in the opened panel “Select node” for a protein of interest (e.g. SPIKE_SARS2). In the maptree, the cluster containing the protein of interest C12-L1 and its ancestor clusters are highlighted in yellow. Finally, by exploding the cluster C12-L1, the subnetwork contained in the cluster is displayed. The subgraph with the protein of interest is highlighted in the red rectangle.

To further validate the predictions made by our system, Table 4 has been created. It reports the list of currently unannotated human proteins, predicted as potential candidate annotations for the functional term GO:0044650 by our tool, and that have been confirmed in the most recent literature works. Only for the human protein SFTPD_HUMAN no clear evidence that supports its association with the functional term considered has been found. Starting from this table and exploiting the “Community-based” option in Fig 6, the protein-protein interaction network is visualized in Fig 9, where the communities containing the proteins predicted as “positive” by our method have already been exploded in order to visualize the entire community.

**Table 4:** List of potential novel associations predicted with a high score and confirmed by the most recent literature works.

UniProt KB-AC	UniProt KB-ID	Description	Score	Literature Evidence
P35247	SFTPD_HUMAN	Surfactant Protein D	0.971	not found
O15393	TMPS2_HUMAN	Transmembrane Serine Protease 2	0.952	[69, 70]
P09958	FURIN_HUMAN	Paired Basic Amino Acid Cleaving Enzyme	0.952	[71, 72]
P35613	BASI_HUMAN	Basigin (Ok Blood Group)	0.917	[73]
Q7Z5G4	GOGA7_HUMAN	Golgin A7	0.917	[14]
Q9C0B5	ZDHC5_HUMAN	Zinc Finger DHHC-Type Palmitoyltransferase 5	0.917	[14, 74]

The information related to the community C12 (the one containing the top scored proteins) have been downloaded (nodes, edges, predictions) to conduct the pathway enrichment analysis, as described in the previous section. The results showed that the KEGG term having the lowest p-value (0.017 using Benjamin-Hochberg correction) is “hsa05164:Influenza A” and includes the following 7 genes: TMPS2_HUMAN, IKKB_HUMAN, RAE1L_HUMAN, NLRP3_HUMAN, TRI25_HUMAN, NUP98_HUMAN, FURIN_HUMAN. Interestingly, this KEGG term contains two of the proteins predicted as ‘positive’ by UNIPred-Web (i.e. FURIN_HUMAN, TMPS2_HUMAN) and related to Spike glycoprotein of viral strain SARS-CoV2. In addition, in the literature we found a clear correlation evidence between Influenza A and SARS-CoV2 [75]. We also found that the other coding genes that turn out to be involved with the selected KEGG term, are also correlated with SARS-CoV2 [14, 70, 76–78].

## Methods and models

### Network integration and protein function prediction

The integration of multiple biological networks for a given organism and protein function consists in providing a consensus network *G* = 〈*V*, *E*〉, embedding information of all individual networks. *V* = {*v*_1_, *v*_2_, …, *v*_*n*_} is the set of proteins, *E* ⊂ *V* × *V* the set of edges, which are associated with a symmetric weight matrix ***W***, with *W*_*ij*_ ∈ [0, 1] denoting the “consensus strength” of connection (*v*_*i*_, *v*_*j*_)∈*E*; moreover, *W*_*ij*_ = 0 if (*v*_*i*_, *v*_*j*_)∉*E*. The integration algorithm already employed by the previous version of the server is UNIPred, an imbalance-aware integration method which obtained competitive results on the MOUSEFUNC I challenge [79] for predicting the function of mouse proteins [9]. The prediction algorithm embedded in UNIPred, COSNet, has been improved in this update by adding a tuning procedure for the *cost* hyper-parameter of COSNet (see [80]), through a two levels grid-optimization procedure, whereas its default value was used in the previous server version. These two algorithms are the base for the realization of task **T1**.

### The hierarchical community detection algorithm

For the identification of the hierarchy of non-overlapping communities (required for the realization of the task **T2**), we have adopted a fast divisive approach that relies on an extended adaptation of the Louvain algorithm [24], which is one of the fastest and the most effective CD algorithms on benchmark evaluations [20]. Furthermore, it is also particularly well-suited to detect meaningful communities on biological networks [81]. Notwithstanding, its direct application is not suitable here, because the hierarchy built after the first phase (vertex-moving) often leads to a large number of communities (mostly singletons), thus limiting the advantages of exploiting a hierarchical visualization. The hierarchy of meta-nodes, instead, constructed in the second phase of the algorithm, might show communities with thousands of nodes, thus making unfeasible their visualization.

To overcome these limitations, we designed a divisive variant of the Louvain algorithm, where it is possible to control the size of communities in the deepest level, in order to obtain sufficiently small communities to be used in our hierarchical multi-resolution visualization. Algorithm 1 contains the pseudocode of our hierarchical CD procedure. At first, the Louvain method is applied to get the set of non-overlapping communities $V_{C}\subset P\left(V\right)$ at level 1 (0 is the root level), that is $\cup_{c\in V_{C}}\;c=V$, and $c\cap \bar{c}=\emptyset$ for each $c,\bar{c}\in V_{C}$ and $c\neq \bar{c}$ (line 1). Here P(V) denotes the power set of *V*. In other words, we kept only the communities obtained in the last step of the Louvain method, the partition of *V* ensuring the highest modularity. It is worth noting that we consider the communities obtained as (meta)nodes in our hierarchy, thus (*V*, *c*) is a directed edge denoting the inclusion relationship of *c* in *V*, and accordingly an edge in our tree, meaning the root *V* is the parent of (meta)node/community *c*.

**Algorithm 1** Divisive hierarchical Louvain algorithm

**Input**: The protein network *G* = (*V*, *E*) *minsplit*, minimum number of nodes to further split a community

**Output**: The community hierarchy *C* = (*V*_*C*_, *E*_*C*_)

1: *V*_*C*_ ← Louvain(*G*)

2: *E*_*C*_ ← ∅

3: *E*_*C*_ ← *E*_*C*_ ∪ {(*V*, *c*)}, ∀*c* ∈ *V*_*C*_

4: *S* ← *V*_*C*_

5: **while** *S* ≠ ∅ **do**

6:  *c* ← Extract(*S*)

7:  **if** GainModularity(*G*_*c*_) **and** |*c*| ≥ *minsplit* **then**

8:   Construct subgraph *G*_*c*_

9:   $\bar{V}\leftarrow \mathrm{Louvain}\left(G_{c}\right)$

10:   $S\leftarrow S\cup \bar{V}$

11:   $V_{C}\leftarrow V_{C}\cup \left\{\bar{V}\right\}$

12:   **for** $\bar{c}\in \bar{V}$ **do**

13:    $E_{C}\leftarrow E_{C}\cup \left\{\left(c,\bar{c}\right)\right\}$

14:   **end for**

15:  **end if**

16: **end while**

17: **return** (*V*_*C*_, *E*_*C*_)

Then, to build up the next levels in the hierarchy tree, to deal with our graphical requirement of having smaller communities to be explored in the browser, and to still exploit the effectiveness of the Louvain method, we first constructed the set of undirected subgraphs *G*_*c*_ induced by the subsets of nodes *c* ∈ *V*_*C*_ (line 8), then applied the Louvain algorithm to each subgraph separately (line 9)—by still keeping only the communities at level 1 of the obtained hierarchy, for the same reasons mentioned above. This produces the level 2 of the tree, where again the communities form a partition of *V*. In lines 5-16, iteratively in a top-down fashion, each subgraph/community is then further split if its size is sufficiently large and the local modularity increases with the split (line 7), otherwise no split is carried out. The while loop at lines 5-16 ends when no community can be further split. For graphical reasons, we have set the minimum number of nodes to split a community (*minsplit*) according to the size of the network (ranging from 50 for the CoV-human network to 350 for the largest ones—more than 15K nodes). We remark that the number of resulting communities in the hierarchy can be large, since it is possible to obtain very small communities. The time complexity of this procedure depends on the complexity of the Louvain algorithm, which can be computed in O(h·E) time when efficiently computing the modularity update, which in practice reduces to O(E), due to the fast convergence behaviour of the algorithm [82]. Our extension iterates the application of the Louvain algorithm down to the leaves on communities that at each level of the tree at most cover the original graph (some communities might not be split due to their size or to no further possible gain in modularity). Thus at each level of the tree, the complexity of the algorithm is still O(E), and O(l·E) for the whole execution, where *l* is the number of levels in the tree. *l* in turn depends on *n* and on the *minsplit* parameter. In practice, we have observed that *l* tends to be much small (often lower than 10), thus making the overall complexity in practice still O(E).

Hereafter, with little abuse of notation for the sake of readability, we denote by *V*_*C*_ the set of communities detected (those at the lower and higher levels of the hierarchy). The resulting hierarchy is structured as a tree *C* = (*V*_*C*_, *E*_*C*_), where we recall *E*_*C*_ ⊂ *V*_*C*_ × *V*_*C*_ represents the inclusion relationship, that is $\left(c,\bar{c}\right)\in E_{C}$ it means that $\bar{c}$ is contained in *c* (lines 3, 13). The reader can refer to the top part of Fig 9 for an example of the hierarchy represented as a maptree. Accordingly, two communities in the tree are *disjoint* when they do not belong to same path to the tree root, and one is *included* in the other one when a path from the former to the root exists that contains the latter. The meaningfulness of the communities detected by this extension of the Louvain method has been experimentally validated in Section *Results*.

### Multi-resolution representation of an integrated network

The obtained community tree *C* allows to expand or compress communities to supply different visualization resolutions needed in task **T3**. On the other side, fixed a resolution level, the view might contain at the same time meta-nodes and individual proteins, and their reciprocal relationships. In particular, connections among two communities denote the presence of at least one edge (in *G*) adjacent to a protein in one community and to a protein in the other community. Whereas, an edge between a single protein *v* and a community means that there exists at least one edge in *G* whose extremes are *v* and one protein in that community. More formally, a view at a given resolution level can be represented as a graph *G*_*L*_ = (*V*_*L*_, *E*_*L*_) where two kinds of nodes can be present: *meta-nodes*, denoted ($V_{L}^{\text{meta}}\subseteq V_{C}$), and representing the communities; and *atomic nodes*, denoted ($V_{L}^{\text{node}}\subseteq V$), and representing biomolecules occurring in the integrated network *G*.

Due to the different types of nodes that are present in *V*_*L*_, three kinds of edges can be identified in *E*_*L*_: those that belong to the integrated network $\left\{\left(v,\bar{v}\right)\in E\;|\;v,\bar{v}\in V_{L}^{\text{node}}\right\}$; those that represent relationships among communities $\left\{\left(c,\bar{c}\right)\;|\;c,\bar{c}\in V_{L}^{\text{meta}}\right\}$, which means that there exist $v,\bar{v}\in V$, with *v* ∈ *c* and $\bar{v}\in \bar{c}$ such that $(v,\bar{v})\in E$; finally, those that represent relationships among biomolecules and communities: $\left\{\left(v,\bar{c}\right)\;|\;v\in V_{L}^{\text{node}},\;\bar{c}\in V_{L}^{\text{meta}}\right\}$, for which there exists $\bar{v}\in \bar{c}$ such that $(v,\bar{v})\in E$. Fig 3 reports three views that can be obtained from the integrated network of our running example. The view on the left-hand side contains only two meta-nodes and an edge between them representing the existence of proteins of the first community that are in relation with proteins of the second community. The view on the right-hand side contains both meta-nodes and proteins. Three kinds of edges can be detected: the dashed line between C1 and C2 represents a relationship between two communities; the dotted line between C5 and the protein SPY represents a relationship between a community and a biomolecule; whereas, the straight lines represent relationships between proteins.

This multi-resolution representation improves the rendering of the network on the screen at different levels of resolution, and highlights the relationships existing among communities and among biomolecules and communities. Moreover, starting from a community in the multi-resolution representation of an integrated network on the screen, the user can interactively decide to move to lower layer communities by opening its content according to the community hierarchy *C* (invoking the zoom_in operation of task **T4**) or to remove details by moving to a higher level community in the hierarchy (invoking the zoom_out operation of task **T5**). In this way the user can analyse and identify properties of the integrated network and point out hidden knowledge on the structure, communities and relationships occurring on the biomolecular network.

### Platform architecture, graph visualization and indexes

The visualization facilities so far discussed need the development of different software components (network visualization, visual interaction, integration and prediction processing, multi-resolution navigation and data storage and processing) and accessing strategies with the aim of smoothly navigating among the communities and easily identifying and retrieving the target proteins in networks with thousand of nodes.

Our software components exchange messages and minimize the amount of data that need to be transmitted. Relying on a Client-Server architecture, that moves the time-consuming operations to the server-side, we achieved very good performances, which are also positively affected by the implementation of many operations directly within the Mysql DBMS by means of stored procedures and the use of indexing structures that makes efficient the identification of edges incident in a node. Mysql, Php, R, Node.js are used for the storage and processing of data on the server side. Javascript, Cytoscape.js, and AngularJS have been used on the client side for the visualization and rendering of the biomolecular networks; these libraries allow the user interaction and network exploration. These technologies are the building blocks on top of which UNIPred-Web works.

To make feasible an efficient and interactive exploration and navigation of a network at different levels of resolution, we developed different indexing structures specifically developed for working with the communities. Each community *c* ∈ *C* is associated with a triple of indexes (*pre*, *post*, *level*) corresponding to the pre-order and post-order visit of *C*, and the level of the community *c* in the hierarchy tree. To streamline the notation, in the remainder *c* will denote, when not expressly remarked, a community or the index associated with it. In addition, each node of the integrated network *G* = 〈*V*, *E*〉 is associated with the pair of indexes (*pre*, *post*), corresponding to the most specific community in which the biomolecule has been included (i.e. a community that is a leaf in the hierarchy *C*). Moreover, the graph is unordered and edges are ordered according to the pre-order indexes of the most specific community of their vertices (i.e., we do not distinguish between (*v*_1_, *v*_2_) and (*v*_2_, *v*_1_), and *pre*(*v*_1_) is always lower than *pre*(*v*_2_)). In this way, according to [83], the following operations can be realized in constant time:

- determine when *c*_1_ is a descendant of *c*_2_ (denoted *c*_1_ ∈ *desc*(*c*_2_)), and the parent of a community *c* (denoted *parent*(*c*)),
- determine the leaf community a protein *v* belongs to (denoted *class*(*v*)).
- determine when two communities *c*_1_, *c*_2_ are disjoint (denoted *c*_1_≁*c*_2_) and meaning that *c*_1_ ∉ *desc*(*c*_2_) and *c*_2_ ∉ *desc*(*c*_1_);
- determine when a protein *v* belongs to a class *c* in the hierarchy *C* (denoted *v* ∈ *c*).

**Algorithm 2** Construction of the index $I_{C}$

**Input**: The community hierarchy *C* = (*V*_*C*_, *E*_*C*_), The protein network *G* = (*V*, *E*)

**Output**: the index $I_{C}$ containing the relationships among the communities in *C* induced by the edges in *G*

*V*_*I*_ ← *V*_*C*_

*E*_*I*_ ← ∅, ${\bar{E}}_{I}=\emptyset$

1: **for each** (*v*_1_, *v*_2_)∈*E* s.t. *class*(*v*_1_)≠*class*(*v*_2_) **do**

2:  ${\bar{E}}_{I}\leftarrow {\bar{E}}_{I}\cup \left\{\left(class\left(v_{1}\right),class\left(v_{2}\right)\right)\right\}$

3: **end for**

4: **for each** (*c*_1_, *c*_2_)∈*V*_*C*_ × *V*_*C*_ s.t. *c*_1_≁*c*_2_, *pre*(*c*_1_)<*pre*(*c*_2_) **and**

5:   $\exists \left(\bar{c_{1}},\bar{c_{2}}\right)\in {\bar{E}}_{I}$ s.t. $\bar{c_{1}}\in desc\left(c_{1}\right)$ and $\bar{c_{2}}\in desc\left(c_{2}\right)$ **do**

6:  *E*_*I*_ = *E*_*I*_ ∪ {(*c*_1_, *c*_2_)}

7: **end for**

8: **return** $\left(V_{I},E_{I}\cup {\bar{E}}_{I}\right)$

Starting from these basic indexing structures, a more complex index has been realized for inducing the relationships existing among non-overlapping communities in *C* by means of the edges *E* of the integrated network. This index (named $I_{C}$) is a graph (*V*_*I*_, *E*_*I*_) whose nodes are the communities in *V*_*C*_ and *E*_*I*_ contains the edges between two non-overlapping communities (*c*_1_, *c*_2_) for which at least an edge exists in *G* among the nodes belonging to the communities *c*_1_ and *c*_2_. The construction of this index is realized in two steps by means of Algorithm 2. In the first step (lines between 1 and 3), the edges that occur among the communities in *V*_*C*_ that are leaves of the hierarchy are determined. This is accomplished by selecting the edges whose source protein and target protein fall in different leaf communities. All the leaf communities of the hierarchy *C* should be considered because they are all disjoint.

In the second step (lines between 4 and 7), the edges with the other communities in the hierarchy *C* are determined by considering all possible pairs $\left(c,\bar{c}\right)$ of disjoint communities (with the exception to those for which *c* and $\bar{c}$ are both leaves). Among them, we include in *E*_*I*_ only the pairs (*c*_1_, *c*_2_) for which an edge $\left(\bar{c_{1}},\bar{c_{2}}\right)$ were included in ${\bar{E}}_{I}$ in the first step of the algorithm such that $\bar{c_{1}}$ (respectively $\bar{c_{2}}$) is a descendant of *c*_1_ (respectively *c*_2_). By following this two-step algorithm all the possible edges between two non-overlapping communities are included in (*V*_*I*_, *E*_*I*_) and can be exploited for the generation of a multi-resolution representation *G*_*L*_. This two-step algorithm requires to use the edges in *E* only in the first step (the complexity of this operation is in O(|E|)) and, in the second step, only disjoint communities in the hierarchy are considered (the complexity of this operation is in $O(|V_{C}|\times |V_{C}|)$).

Since all the operations for checking when an edge belongs to a generic community in *C* are executed in constant time, the complexity of the process for inducing the relationships existing among non-overlapping communities is in $O(|E|+|V_{C}|\times |V_{C}|)$. Usually, the communities that are identified for an integrated network are much lesser than the nodes in *V* and the edges in *E*, therefore the previous formula can be simplified as O(|E|).

**Algorithm 3** Zoom in

**Input**: the index $I_{C}=\left(V_{I},E_{I}\right)$,

   the multi-resolution representation *G*_*L*_ = (*V*_*L*_, *E*_*L*_)

   a community *c* ∈ *V*_*L*_

   the community hierarchy *C* = (*V*_*C*_, *E*_*C*_),

   the protein network *G* = (*V*, *E*)

**Output**: a new $G_{L}^{\prime }=\left(V_{L}^{\prime },E_{L}^{\prime }\right)$ with the node *c* expanded

1: $V_{L}^{\text{node}}\left(c\right)\leftarrow \left\{v|v\in V_{L}^{\text{node}}\wedge \left(v,c\right)\in E_{L}\right\}$

2: $V_{L}^{\text{meta}}\left(c\right)\leftarrow \left\{v|v\in V_{L}^{\text{meta}}\wedge \left(v,c\right)\in E_{L}\right\}$

3: **if** *c* is a leaf community in *C* **then**

4:  *V*(*c*)←{*v* ∈ *V*|*class*(*v*) = *c*}

5:  *E*(*c*)←{(*v*_1_, *v*_2_)|(*v*_1_, *v*_2_)∈*E*∧*v*_1_, *v*_2_ ∈ *V*(*c*)}

6:  $E_{L}^{new}\leftarrow \left\{\left(v_{1},v_{2}\right)|v_{1}\in V\left(c\right),v_{2}\in V_{L}^{\text{node}}\left(c\right),\left(v_{1},v_{2}\right)\in E\right\}\cup \{\left(v_{1},c_{2}\right)|v_{1}\in V\left(c\right),c_{2}\in V_{L}^{\text{meta}}\left(c\right),\exists v^{\prime }\in c_{2}\;\text{s.t.}\;(v_{1},v\prime )\in E\}$

7: **else**

8:  *V*(*c*)←{*c*′ ∈ *V*_*C*_|(*c*, *c*′)∈*E*_*C*_}

9:  *E*(*c*)←{(*c*_1_, *c*_2_)|(*c*_1_, *c*_2_)∈*E*_*I*_∧*c*_1_, *c*_2_ ∈ *V*(*c*)}

10:  $E_{L}^{new}\leftarrow \left\{\left(c_{1},v_{2}\right)\right|c_{1}\in V\left(c\right),v_{2}\in V_{L}^{\text{node}}\left(c\right),\exists v^{\prime }\in c_{1}\;\text{s.t.}\;(v\prime ,v_{2})\in E\}\cup \{\left(c_{1},c_{2}\right)|c_{1}\in V\left(c\right),c_{2}\in V_{L}^{\text{meta}}\left(c\right),\left(c_{1},c_{2}\right)\in E_{I}\}$

11: **end if**

12: $V_{L}^{\prime }=V_{L}\cup \left(V\left(c\right)\backslash \left\{c\right\}\right)$

13: $E_{L}^{\prime }=\left(E_{L}\backslash \left\{\left(v,c\right)|\left(v,c\right)\in E_{L}\right\}\right)\cup E_{L}^{new}\cup E\left(c\right)$

14: **return** $\left(V_{L}^{\prime },E_{L}^{\prime }\right)$

### Initial multi-resolution graph and zoom_in/zoom_out operations

The initial multi-resolution representation of an integrated network is formed by a single meta-node (the root of the hierarchy *C*) and no edges are present (i.e. *G*_*L*_ = ({*c*}, ∅), where *c* = *root*(*C*). The user can thus apply the zoom_in operation for enlarging the visualization starting from the meta-node *c*. Besides this very particular situation, *G*_*L*_ is composed by proteins and metanodes, and the user can ask to apply the zoom_in operation on any metanodes, or he/she can apply the zoom_out operation on proteins or metanodes. The effect is to produce a new multi-resolution representation of the integrated graph in which metanodes are expanded. Algorithm 3 reports the pseudo-code of the zoom_in operation. Starting from the current *G*_*L*_, the community hierarchy *C*, the index $I_{C}$, the integrated network *G* = (*V*, *E*) and the community *c* to be expanded, it allows us to create a new multi-resolution representation $G_{L}^{\prime }$ in which the community *c* and the edges that connect *c* with other communities/proteins in *G*_*L*_ are substituted with the communities/proteins contained in *c*. First the algorithm identifies the communities $V_{L}^{\text{meta}}\left(c\right)$ and the proteins $V_{L}^{\text{node}}\left(c\right)$ that are incident with *c* in *G*_*L*_ (lines 1 and 2). When *c* is a leaf node of the community hierarchy *C*, *c* needs to be substituted with its proteins. Therefore, the proteins of the community *c*, i.e. *V*(*c*), and all internal edges in the community, i.e. *E*(*c*), are determined (lines 4 and 5). At this point, the edges between the subgraph (*V*(*c*), *E*(*c*)) and the vertices in $V_{L}^{\text{node}}\left(c\right)$ are determined by exploiting the edges in *G*, whereas the edges between the subgraph (*V*(*c*), *E*(*c*)) and the vertices $V_{L}^{\text{meta}}\left(c\right)$ are determined by identifying edge between the nodes in *V*(*c*) and the proteins in *V* associated with the communities in $V_{L}^{\text{meta}}\left(c\right)$ (line 6). When *c* is an aggregated community of the community hierarchy *C*, *c* needs to be substituted with its child communities. Therefore, the communities *V*(*c*) that are children of *c* in *C* are determined (line 8). The new edges to be included in $G_{L}^{\prime }$ are determined in two steps: first, we determine through the index $I_{C}$ the existing edges among the nodes in *V*(*c*); then, we determine the edges between the proteins associated with the communities in *V*(*c*) and the proteins in $V_{L}^{\text{node}}\left(c\right)$ and the edges between the communities in *V*(*c*) and the communities in $V_{L}^{\text{meta}}\left(c\right)$ that are available in the index structure (lines 10). In both cases, the vertices of $G_{L}^{\prime }$ are obtained by removing from *V*_*L*_ the community *c* and including the vertices in *V*(*c*). The edges of $G_{L}^{\prime }$ are obtained by removing from *E*_*L*_ all the edges that are incident in *c* and including the edges in $E_{L}^{\text{new}}$ and *E*(*c*).

Many of the described operations require to consider the edges *E* that are present in the integrated network *G* and to check properties on them. Since the complexity of the operations for checking the properties is always constant by using our indexing structures, the complexity of the zoom_in operation in the worst case is in O(|E|).

**Algorithm 4** Zoom out

**Input**: the multi-resolution representation *G*_*L*_ = (*V*_*L*_, *E*_*L*_) *c* ∈ *V*_*L*_\{*root*(*C*)}

   the community hierarchy *C* = (*V*_*C*_, *E*_*C*_),

**Output**: a new $G_{L}^{\prime }=\left(V_{L}^{\prime },E_{L}^{\prime }\right)$ with the node *c* collapsed

1: **if** $c\in V_{L}^{\text{node}}$ **then**

2:  *c*_*p*_ ← *class*(*c*)

3:  $V_{L}\left(c_{p}\right)\leftarrow \left\{v\in V_{L}^{\text{node}}|class\left(v\right)=c_{p}\right\}$

4: **else**

5:  *c*_*p*_ ← *parent*(*c*)

6:  $V_{L}\left(c_{p}\right)\leftarrow \left\{c^{\prime }\in V_{L}^{\text{meta}}|\left(c_{p},c^{\prime }\right)\in E_{C}\right\}$

7: **end if**

8: $E_{L}^{new}\leftarrow \{\left(c_{p},v_{1}\right)|v_{1}\in V_{L}\backslash V_{L}\left(c_{p}\right)\wedge \exists v^{\prime }\in V_{L}\left(c_{p}\right)\left|\left(v_{1},v^{\prime }\right)\in E_{L}\right\}$

9: $V_{L}^{\prime }=\left(V_{L}\cup \left\{c_{p}\right\}\right)\backslash V_{L}\left(c_{p}\right)$

10: $E_{L}^{\prime }=E_{L}^{new}\cup \left(E_{L}\backslash \left\{\left(v_{1},v_{2}\right)\in E_{L}|v_{1}\in V_{L}\left(c_{p}\right)\vee v_{2}\in V_{L}\left(c_{p}\right)\right\}\right)$

11: **return** $\left(V_{L}^{\prime },E_{L}^{\prime }\right)$

Algorithm 4 reports the pseudo-code of the zoom_out operation. This operation is simpler than the zoom_in operation because it works only on the current multi-resolution representation *G*_*L*_ by taking into account the community hierarchy *C* and the node *c* that needs to be collapsed. When *c* is a protein node, this operation has the purpose to remove from *G*_*L*_ the protein *c* and also all the other proteins of the same class of *c*. Therefore, at line 3, the set of proteins *V*_*L*_(*c*_*p*_) of class *c*_*p*_ = *class*(*p*) are determined. Whenever, *c* is a meta-node, the zoom_out operation has the purpose to remove from *G*_*L*_ the communities that are children of the parent community of *c* (*parent*(*c*_*p*_)). Therefore, at line 6, the set of metanodes *V*_*L*_(*c*_*p*_) belonging to the class *parent*(*c*_*p*_) is determined. Starting from *V*_*L*_(*c*_*p*_), we are able to determine both the edges in *E*_*L*_ to be removed (the edges that are incident in at least a node in *V*_*L*_(*c*_*p*_)) and those to be included (the edges that substitute an edge (*v*_1_, *v*_2_) between *V*_*L*_\*V*_*L*_(*c*_*p*_) and *V*_*L*_(*c*_*p*_) with an edge (*v*_1_, *c*_*p*_)). Many operations require to consider the edges *E*_*L*_ that are present in *G*_*L*_ and to check properties on them. Also in this case, the checking of the properties can be realized in constant time, then the complexity of the zoom_out operation is in $O(|E_{L}|)$. In the worst case, the size of *E*_*L*_ is *E*, therefore the complexity of the zoom_out operation is in O(|E|).

## Discussion

The novel algorithms and visualization tools proposed in this work introduce the possibility to explore large and complex networks by means of a hierarchy of protein communities, that allows a multi-resolution and interactive visualization and analysis of gene and protein networks. We showed that through our methodology integrated in UNIPred-Web, we are able to visualize and explore protein networks of different size and connectivity. In particular, in experiment n. 2963 (see S1 File) a network having 17287 and 840950 edges is visualized, through a three level hierarchy; whereas through the experiment n. 2964, we efficiently navigated a deep hierarchy of dense communities, having 1910239 edges for 13535 proteins grouped in 265 communities and 7 levels.

As discussed in the comparison with other similar tools, UNIPred-Web does not require specific computational resources and/or devices, and can be run on standard browsers and off-the-shelf machines. Furthermore, the analysis performed on two case studies to predict the GO term p38MAPK cascade exploiting two integrated pathway interaction networks, and to predict *adhesion of symbiont to host cell* term in the CoV-human network, has shown the novel possibilities opened by our tool. Indeed, through the facility for downloading the proteins forming a given community, we can further study the individual communities: for instance we found that community proteins are enriched in meaningful and coherent pathways, highlighting a predominant biological function. Thus, the dissection of networks in communities can help the biologist to pinpoint interesting biological functions needed to plan further *in vitro* studies. Thanks to the novel community-based exploration, in the case study involving the CoV-human network, we have found that two proteins predicted by our tool as positive for the GO term *adhesion of symbiont to host cell* (GO:0044650), namely FURIN_HUMAN and TMPS2_HUMAN, are contained in the KEGG pathway “hsa05164:Influenza A”, resulted enriched with respect to the community containing the top-scored proteins. Interestingly, it is known that Influenza A and SARS-CoV2 are strictly related [78]. Moreover we found novel human proteins that could potentially interact with SARS-CoV2 proteins.

We plan to improve the system along several directions. On the user interface side, one potential extension consists in the integration of external services, like for example CTD—Comparative Toxicogenomics Database, and HPO—The Human Phenotype Ontology [84] for enhancing the information associated with proteins, and thus further supporting the users in conducting their investigations. Moreover, on the algorithmic side, although the already implemented methods provide state-of-the-art performance in predicting protein functions [9], the prediction engine will be constantly improved in light of novel research results. From the results analysis side, state-of-the-art methods for enrichment analysis could also be embedded in the system, to provide relevant complementary information about proteins and their biomolecular functions directly integrated within the tool. A further development is the application of the visualization facilities here described to manage networks of patients [85], in order to visualize and manage communities of patients to stratify them according to their biomolecular profiles.

## Supporting information

S1 File Data availability and experiment codes for testing the application. (PDF)
