# Supplementary material for: Multi-resolution visualization and analysis of biomolecular networks through hierarchical community detection and web-based graphical tools
Source: PLoS One. 2020 Dec 22;15(12):e0244241. doi: 10.1371/journal.pone.0244241 (PMC7755227; doi:10.1371/journal.pone.0244241)
Supplement: S1 File — (PDF) [file pone.0244241.s001.pdf]

S1 File: Multi-resolution visualization and analysis of biomolecular networks through hierarchical community detection algorithms and web-based graphical tools

Paolo Perlasca[1], Marco Frasca[1], Cheick Tidiane Ba[1], Jessica Gliozzo[2], Marco Notaro[1], Mario Pennacchioni[1], Giorgio Valentini[1,3], Marco Mesiti[1,*]

1 Department of Computer Science, Università degli Studi di Milano, Milano, Italy
2 Unit of Neuroradiology, IRCCS Ospedale San Raffaele, Milano, Italy
3 CINI National Laboratory in Artificial Intelligence and Intelligent Systems - AIIS, Roma, Italy
* mesiti@di.unimi.it

Characteristics of the UNIPred-Web application

UNIPred-Web website: `http://unipred.di.unimi.it`

Operating system: Platform independent

Supported Browsers: The Web application has been tested in several Operating systems (Windows 10, Mac OS Yosemite, Ubuntu 18 LTS) with the following browsers: FIREFOX (version 65.0 64bit), CHROME (version 73.0.3683.103 64 bit). On these browsers the application works perfectly without issues. The application works also well with Safari 10.1.22 on Mac OS Yosemite. We have also considered EDGE (version 44.17763.1.0) on Windows 10 and it works well with the exception of image download (which is not supported).

Server-side Programming languages: php (version 5), node.js (version 10.1.0), R-scripts (version 3.5)

Client-side Programming languages: angular.js (version 1.2.16), cytoscape (version 3), jquery (version 2.2.4)

Database: MariaDB 10

License: GNU GPL

Any restrictions to use by non-academics: none

The datasets are made available through the UNIPred-Web application. Interested users can download the datasets directly from the application. For any concerns, the reader can make a request to the corresponding author.

Experiments codes for testing the Community-based navigation facility

We here provide some use-cases that can be exploited for testing the graphical facilities that we have developed for the community-based navigation of integrated networks. The user can use the experiment code reported below in the load button in the right top corner of the main interface.

Suppose that a cancer research laboratory is interested in investigating novel therapeutic approaches to Glioblastoma Multiforme (GBM) by studying the p38 MAPK signaling pathway, which is known to be dysregulated in gliomas ([1], [2]) and a pivotal target for new drugs ([3], [4], [5]). With this goal in mind, the biologist can exploit the UNIPred-Web functionalities to investigate the considered pathway from different points of views, e.g. creating different integrated networks that highlight complementary aspects and effects of the biological pathway under study. Specifically, the biomedical researcher can make the experiments schematically described as follows.

- Experiment code `2962`. In this experiment we integrated the following networks: Pathway.CELL_MAP [6] and Pathway.REACTOME ([7]). The underlying idea is to enrich the extended metabolic map provided by REACTOME with a network more tailored towards cancerous mechanisms, thus providing a wider spectrum of possible altered pathways. The final integrated network presents 2 380 nodes and 25 501 edges. A 4 levels hierarchy has been generated with a total of 25 communities.
- Experiment code `2963`. To take into account the correlation between gene expression profiles, the researcher has the possibility to choose among a wide list of co-expression networks already integrated in UNIPred-Web, spanning many disease states. In our case scenario, the selection of Co-expression.Ramaswamy-Golub-2001 ([8]) and Co-expression.Lee-Fine-2006 ([9]) is meaningful since the former considers GBM and other 13 different cancers and the latter is specific for GBM. The integrated network presents 17 287 nodes and 840 950 edges. A 3 levels hierarchy has been generated with a total of 158 communities.
- Experiment code `2964`. Here we integrated the Pathway.Wu-Stein-2010 network [10] (already included in our web server) with the user-defined glioma co-expression network taken from [11]. It is worth noting that the latter network is represented by a weighted symmetric adjacency matrix and can be directly uploaded in our web server. Since Pathway.Wu-Stein-2010 was successfully used to study GBM ([10]), the investigator has the opportunity to study a rich cancer network with a consistent amount of proteins and PPI already implicated in the tumor. The final integrated network has 13 535 nodes and 1 910 239 edges. A 7 levels hierarchy has been generated with a total of 265 communities.

The interested user can exploit the experiment code to load the experiments depicted above through the UNIPred-Web server and explore the integrated network shown by means of a community-based visualization. Thanks to the combination of community-based and vertex-centric visualization, the user can have at the same time both a “bird’s eye” and a “magnified” overview of the underlying integrated network. By exploding a community and by properly selecting a network layout among those available, the user is also able to visualize the closest neighbourhood of a specified protein of interest. Finally, it is worth noting that UNIPred-Web scales gracefully with the size of the input networks. Indeed, it can process in a few minutes networks composed of thousands of nodes and hundreds of thousands of edges and it can process

in a reasonable time networks encompassing hundreds of thousands of nodes and millions of edges. More precisely, UNIPred-Web requires about 2, 10 and 30 minutes to integrate the networks used respectively in the first experiment (code `2962`), in the second experiment (code `2963`) and in the third one (code `2964`). During UNIPred-Web processing, the user can either load and explore networks achieved by previous experiments (by using the option Load) or can perform other integration and/or prediction tasks (by opening a new tab).

References

1. Pandey V, Bhaskara VK, Babu PP. Implications of mitogen-activated protein kinase signaling in glioma. Journal of Neuroscience Research. 2016;94(2):114–127. doi:10.1002/jnr.23687.

2. Chen X, Hao A, Li X, Ye K, Zhao C, Yang H, et al. Activation of JNK and p38 MAPK Mediated by ZDHHC17 Drives Glioblastoma Multiforme Development and Malignant Progression. Theranostics. 2020;10:998–1015. doi:10.7150/thno.40076.

3. Yao YQ, Ding X, Jia YC, Huang CX, Wang YZ, Xu YH. Anti-tumor effect of β-elemene in glioblastoma cells depends on p38 MAPK activation. Cancer Letters. 2008;264(1):127 – 134. doi:doi.org/10.1016/j.canlet.2008.01.049.

4. Yeung Y, Bryce N, Adams S, Braidy N, Konayagi M, McDonald K, et al. P38 MAPK inhibitors attenuate pro-inflammatory cytokine production and the invasiveness of human U251 glioblastoma cells. Journal of Neuro-Oncology. 2012;109(1):35–44. doi:10.1007/s11060-012-0875-7.

5. Li Q, Miao Z, Wang R, Yang J, Zhang D. Hesperetin Induces Apoptosis in Human Glioblastoma Cells via p38 MAPK Activation. Nutrition and Cancer. 2019;72:1–8. doi:10.1080/01635581.2019.1638424.

6. Krogan NJ, Lippman S, Agard DA, Ashworth A, Ideker T. The cancer cell map initiative: defining the hallmark networks of cancer. Molecular cell. 2015;58(4):690–698. doi:10.1016/j.molcel.2015.05.008.

7. Fabregat A, Jupe S, Matthews L, Sidiropoulos K, Gillespie M, Garapati P, et al. The Reactome Pathway Knowledgebase. Nucleic Acids Research. 2017;46(D1):D649–D655. doi:10.1093/nar/gkx1132.

8. Ramaswamy S, Tamayo P, Rifkin R, Mukherjee S, Yeang CH, Angelo M, et al. Multiclass cancer diagnosis using tumor gene expression signatures. Proceedings of the National Academy of Sciences. 2002;98. doi:10.1073/pnas.211566398.

9. Lee J, Kotliarova S, Kotliarov Y, Li A, Su Q, Donin NM, et al. Tumor stem cells derived from glioblastomas cultured in bFGF and EGF more closely mirror the phenotype and genotype of primary tumors than do serum-cultured cell lines. Cancer Cell. 2006;9(5):391 – 403. doi:10.1016/j.ccr.2006.03.030.

10. Wu G, Feng X, Stein L. TA human functional protein interaction network and its application to cancer data analysis. Genome Biol. 2010;11(R53). doi:10.1186/gb-2010-11-5-r53.

11. Lee S, Zhang C, Arif M, Liu Z, Benfeitas R, Bidkhori G, et al. TCSBN: a database of tissue and cancer specific biological networks. Nucleic Acids Research. 2017;46(D1):D595–D600. doi:10.1093/nar/gkx994.
